# Social media addiction and borderline personality disorder: a survey study

**DOI:** 10.3389/fpsyt.2024.1459827

**Published:** 2025-01-08

**Authors:** Madison Collins, Jon E. Grant

**Affiliations:** Department of Psychiatry & Behavioral Neuroscience, University of Chicago, Pritzker School of Medicine, Chicago, IL, United States

**Keywords:** social media addiction, behavioral addictions, personality, borderline personality, comorbidity

## Abstract

**Background and aims:**

Borderline personality disorder (BPD) is a serious and difficult to treat psychiatric condition characterized by affective and interpersonal instability, impulsivity, and self-image disturbances. Although the relationship between BPD and substance use disorders has been well-established, there has been considerably less research regarding behavioral addictions in this population. The purpose of this study is to determine the prevalence of social media addiction (SMA) among individuals with BPD and to explore whether it is related to aspects of disorder symptomology.

**Methods:**

300 adults completed an online survey via Prolific. Individuals completed the McLean Screening Instrument for BPD (MSI-BPD), along with the Bergen Social Media Addiction Scale (BSMAS). Additionally, all participants reported how often they use social media for the following reasons: distraction from interpersonal problems, reassurance seeking, self-confidence issues, and anger/revenge.

**Results:**

Of the 289 subjects that completed all measures, 38 (13.1%) screened positive for BPD. Individuals screening positive for BPD were more likely to meet criteria for SMA than controls, and they were more likely to report using social media for interpersonal distraction, reassurance seeking, self-confidence issues, and anger/revenge seeking than controls. Among individuals with BPD, SMA was positively associated with the frequency of each of these behaviors, except for anger/revenge seeking.

**Discussion and conclusion:**

The results of this study demonstrate that SMA is common among the BPD population and may be related to aspects of disorder symptomology. Whether SMA worsens BPD symptoms or whether addressing SMA could lead to improvements in the BPD remains to be seen and is an important area for future research.

## Introduction

Borderline personality disorder (BPD) is a serious and difficult-to-treat psychiatric condition characterized by affective and interpersonal instability, self-image disturbances, anger and aggression, suicidality, and impulsivity (1, 2). Epidemiological studies have estimated the prevalence rate of BPD to be around 2.7 to 5.8%, making this disorder relatively common in the general population (1, 3). BPD creates a psychological toll that can drastically reduce quality of life and is also associated with substantial economic and societal burden (4, 5).

Individuals with BPD frequently present with psychiatric comorbidities such as major depressive disorder, post-traumatic stress disorder, bipolar disorder, and anxiety disorders (6–9). However, perhaps the most common psychiatric comorbidity reported in the literature is substance use disorders (SUDs). Rates of comorbid SUDs vary by study, with current prevalence rates ranging from 13% to 73% and lifetime prevalence rates ranging from 45% to 86% (10, 11).

While the term “addiction” has traditionally referred to dependence on psychoactive substances such as alcohol or cocaine, there has been a growing recognition that certain *behaviors* may also become addictive (i.e., “behavioral addictions”) (12, 13). Research has shown that behavioral addictions share may similarities to substance addictions: both are characterized by the pursuit of a behavior despite knowledge of its harmful effects, tension before performing the behavior and relief afterwards, along with tolerance to the behavior (12). Additionally, behavioral addictions share common neurobiological and neurocognitive features with substance addictions, and they often co-occur in the same individual (12).

Perhaps since the study of behavioral addictions is relatively new, these conditions have not been studied in BPD as extensively as SUDs have been. However, both Bagby et al. (14) and Sacco et al. (15) found that individuals with BPD were more likely to meet criteria for problematic gambling/gambling disorder relative to individuals without this personality pathology. Similarly, Maraz et al. (16) found that individuals with BPD were nearly five times more likely to meet criteria for compulsive buying disorder, and Lu et al. (17) found that BPD symptoms were positively associated with internet addiction severity. This limited body of research suggests that behavioral addictions may also be common among individuals BPD, but there remains much more to be explored in this area.

For example, due to the recent rise of social media, researchers have begun to investigate problematic social media use, or social media addiction (SMA). The term remains controversial in the literature and is yet to be classified an official disorder, but it broadly refers to maladaptive use of social media that shares the core feature of addiction: salience (thinking about social media frequently), tolerance (needing to spend more time on social media), mood modification (using social media to alter affect), relapse (being unable to refrain from social media use), withdrawal (negative mood when prevented from using social media), and conflict (interpersonal difficulties stemming from social media use) (18). Prevalence rates of SMA vary by study, but estimates have ranged from 3% to 23% (19–21). Research has shown that SMA is associated with negative mental health outcomes such as depression, anxiety, and loneliness (19, 22, 23), but no research to our knowledge has examined SMA in personality pathologie*s* such as BPD.

Examining SMA in BPD is interesting for several reasons. First, due to the nature of BPD symptomology, SMA has the potential to be highly prevalent in this population. Individuals with BPD often have a need for social approval and a fear of abandonment, which may lead to excessive posting on social media and using it to check on the whereabouts of the important others. In fact, Ooi et al. (24) found that individuals with BPD traits posted on social media sites more frequently and ascribed more importance to social media in their daily lives than individuals without BPD traits. Additionally, individuals with BPD are often impulsive, which has been associated with problematic social media use in several studies (see 25 for a review).

Next, in addition to being prevalent among the BPD population, SMA may also be related to disorder symptomatology. For instance, because social media affords people the opportunity to stay connected with family and friends, individuals with BPD may use these platforms to facilitate the excessive reassurance seeking and attempts to avoid abandonment that are hallmark of this disorder (26). Furthermore, individuals with BPD often have low self-esteem or lack a clear sense of self (27) and may therefore turn to social media as an attempt to ameliorate some of these concerns. Moreover, anger and aggression are common symptoms of BPD, which are typically reactive in nature and result from interpersonal difficulties or perceived threat (28). It is quite common for individuals, including those with BPD, to use social media for cyberbullying or cyberaggression purposes (29–31), and research has shown that problematic social media use is associated with cyberbullying perpetration (30). As such, it may be possible that social media becomes a medium through which anger and frustration are expressed for individuals with BPD, and that SMA increases this tendency.

The current study seeks to contribute to the literature regarding behavioral addictions in BPD by examining the prevalence of SMA in those with BPD relative to those without. Additionally, to capture possible associations between SMA and BPD symptomology, we will examine whether SMA is related to the use of social media for any of the following reasons: reassurance seeking, distraction from interpersonal problems, self-confidence issues, and anger/aggressive outbursts. Based on the existing literature, we predicted that SMA would be more prevalent in those with BPD relative to those without the condition. Moreover, among those with BPD, we predicted that SMA would be positively associated with using social media for reassurance seeking, interpersonal distraction, self-confidence issues, and anger/aggressive outbursts.

## Methods

### Participants

300 adults (52.7% women), aged 19 to 76, were recruited to complete an online survey via Prolific. Interested subjects were told that they would be participating in a study about online behaviors, personality, and mental health. Inclusion criteria for the study were: a) 18 years of age or older; b) currently residing in the United States; c) ability to understand English; and d) capable of providing informed consent. Subjects were excluded if they were unable to understand or undertake in study procedures.

### Procedures

Subjects completed an online survey via REDCap as part of this study. Subjects were first required to view Institutional Review Board (IRB) – approved consent page, at which point they could choose to participate in the survey or opt out. A refusal to respond was taken as a denial of consent and subjects were not allowed to continue with the study. The survey asserted that all responses would be kept confidential and that no personally identifying information would be collected. Subjects were compensated $12 for their participation. Data was collected on 01/09/24.

### Measures

The online survey collected information regarding demographic characteristics, mental health history (subjects were asked to select all psychiatric conditions they had been diagnosed with) and current use of social media platforms (e.g., Instagram, Facebook, TikTok, etc.).

To assess for BPD, all subjects completed the McLean Screening Instrument for Borderline Personality Disorder (MSI-BPD) (32) The MSI-BPD consists of ten questions that individuals respond “yes” or “no” to (e.g., “have your closest relationships been troubled by a lot of arguments or repeated breakups?”). Answering “yes” to seven or more questions (i.e., scoring 7 or higher) is deemed to be the clinical threshold for BPD (32). The MSI-BPD showed good reliability in the present study (α = 0.84).

Subjects completed the Bergen Social Media Addiction Scale (BSMAS) (33), which a commonly used and well-validated measure of SMA. The BSMAS is based on the component model of addiction and includes items that capture each of the six facets. Subjects are asked to respond to each statement on a scale of 1 = Very Rarely, to 5 = Very Often (e.g., “You feel an urge to use social media more and more”). Higher scores indicate greater dependence on social media, and Bányai et al. (34) proposed a cutoff score of 19 (out of 30) as indicating the presence of SMA. The BSMAS showed good reliability in the present study (α = 0.87).

To capture potential manifestations of BPD-like behaviors on social media, all subjects were asked to indicate how often they engage in the following behaviors on a scale of 1 = Never to 4 = Always: “use online platforms (e.g., dating apps, social media) to distract yourself from interpersonal problems you are having”; “turn to social media when you have problems with your self-esteem or confidence”; “use social media and/or dating apps to reassure yourself that people still care about you”; and, “use social media to lash out or get revenge on people that have wronged you”.

### Statistical analysis

The percentage of subjects who met criteria for BPD based on the MSI-BPD was determined. Between-group differences in demographic and clinical characteristics were tested using Pearson chi-square or Fisher’s Exact tests for categorical variables, and independent samples *t*-tests for continuous variables. Between-group differences in total BSMAS score was calculated using an independent samples *t*-test. A Fisher’s exact test was used to determine if there is a difference in rates of SMA (classified as a total BSMAS score of ≥ 19) between individuals screening positive for BPD and controls.

As the questions assessing the use of social media for BPD-like behaviors were answered on an ordinal Likert scale, non-parametric Mann Whitney U tests were used to 1) examine differences between those screening positive for BPD and controls and 2) among those with BPD, differences between those meeting criteria for SMA vs. those who did not. RStudio Version 1.3.1056 was used for all data analysis.

### Ethics

The Institutional Review Board of the University of Chicago approved the study and the consent statement. The authors assert that all procedures contributing to this work comply with the ethical standards of the relevant national and institutional committees on human experimentation and with the Helsinki Declaration of 1975, as revised in 2008.

## Results

### Sample description

A total of 300 individuals completed the online survey via Prolific. 11 subjects did not complete the MSI-BPD screen and were therefore not included in the analysis. As such, the final sample consisted of 289 adults, ages 19 to 76 (M_age_ = 37.38, SD = 12.60).

A total of 38 (13.1%) individuals screened positive for BPD based on the MSI-BPD (i.e., a score of 7 or greater on this scale). All other subjects were included in the control group. Demographic and clinical characteristics of the sample are presented in **Table 1**. An independent samples *t-*test revealed that individuals screening positive for BPD were significantly younger (M = 33.2, SD = 11.79) than individuals in the control group (M = 38.1, SD = 12.62) (*t*(284) = 2.25, *p* = .025). Additionally, individuals screening positive for BPD were more likely to self-report a diagnosis of depression (52.6% vs. 30.7%) (*X* ^2^(1) = 7.13, *p* = .008) or PTSD (21.0% vs. 9.6%) (*p* = .049) than individuals in the control group.

**Table 1:** Demographic and clinical characteristics of sample^a^.

	Total (N = 289)	BPD (N = 38)	Controls (N = 251)	*p*-value
Age, years (mean, SD)	37.48 (12.60)	33.2 (11.79)	38.1 (12.62)	.03^b^
Gender				.29^c^
Male	120 (41.5)	15 (39.4)	105 (41.8)	
Female	154 (53.2)	21 (55.3)	133 (53.0)	
Transgender	4 (1.4)	0 (0)	4 (1.6)	
Other	5 (1.7)	2 (5.3)	3 (1.2)	
Not Reported	6 (2.1)	0 (0)	6 (2.4)	
Race				.14^d^
White/Caucasian	195 (67.5)	24 (63.2)	171 (68.1)	
Black/African American	41 (14.2)	7 (18.4)	34 (13.5)	
Hispanic/Latino	15 (5.2)	1 (2.6)	14 (5.6)	
Asian/Pacific Islander	10 (3.5)	1 (2.6)	9 (3.6)	
American Indian/Alaskan Native	2 (0.7)	1 (2.6)	1 (0.4)	
Multiple Races/Other	21 (7.2)	3 (7.9)	18 (7.2)	
Not Reported	5 (1.7)	1 (2.6)	4 (1.6)	
Education				.14^d^
Some High School	4 (1.4)	0 (0)	4 (1.6)	
High School Degree	35 (12.1)	10 (26.3)	25 (10.0)	
Some College	75 (26.0)	10 (26.3)	65 (25.9)	
College Degree	135 (46.7)	16 (42.1)	119 (47.4)	
Masters Degree	33 (11.4)	2 (5.3)	31 (12.4)	
Professional/Doctoral Degree	4 (1.4)	0 (0)	4 (1.6)	
Not Reported	3 (1.0)	0 (0)	3 (1.2)	
Psychiatric Conditions^e^				
ADHD	39 (13.5)	8 (21.1)	31 (12.4)	.23^c^
Alcohol Use Disorder	7 (2.4)	1 (2.6)	6 (2.4)	1^d^
Autism Spectrum Disorder	4 (1.4)	0 (0)	4 (1.6)	1^d^
Bipolar Disorder	12 (4.2)	3 (7.9)	9 (3.6)	.20^d^
Depression	97 (33.6)	20 (52.6)	77 (30.7)	.008^c^
Generalized Anxiety Disorder	79 (27.3)	13 (34.2)	66 (26.3)	.31^c^
Obsessive Compulsive Disorder	17 (5.9)	4 (10.5)	13 (5.2)	.26^d^
PTSD	32 (11.1)	8 (21.0)	24 (9.6)	.049^d^
Substance Use Disorder	7 (2.4)	1 (2.6)	6 (2.4)	1^d^

a Data are presented as N (%) unless otherwise specified.

b Statistical test: Independent samples *t*-test.

c Statistical test: Chi-square.

d Statistical test: Fisher exact.

e Based on self-report data.

For both the BPD and control groups, approximately 97% of subjects reported current use of one or more social media platforms. Information about the most commonly reported social media platforms for both groups can be found in **Supplementary Appendix A**.

### BPD vs. healthy controls

An independent samples *t*-test revealed that individuals who screened positive for BPD scored higher on the BSMAS (M = 15.6, SD = 5.53) than individuals in the control group (M = 11.9, SD = 4.60) (*t*(274) = -4.48, *p* <.001). Similarly, a Fisher’s Exact test revealed that individuals who screened positive for BPD were more likely to meet the clinical cutoff for SMA than controls (*p* = .02). Specifically, 25% of individuals screening positive for BPD met the threshold for SMA, relative to only 9.6% of individuals in the control group.

Additionally, Mann Whitney U tests revealed that individuals screening positive for BPD were more likely to use social media for distraction from interpersonal problems (*U* = 2705, *p* <.001), self-confidence issues (*U* = 2988, *p* <.001), reassurance seeking (*U* = 3343.5, *p* <.001), and anger/revenge (*U* = 4085, *p = .*007) than individuals in the control group.

### BPD only

Only subjects who screened positive for BPD were included in the following analyses. Of the 38 subjects who screened positive for BPD, 37 (97.4%) of them reported current use of social media. Demographic and clinical characteristics of the subjects who reported current social media use are presented in **Table 2**. There were no differences in age, gender, race, education, or self-reported psychiatric conditions between those who met criteria for SMA and those who did not.

**Table 2:** Demographic and Clinical Differences Between BPD Subjects with SMA vs. Those Without^a^.

	BPD + SMA (N = 9)	BPD Only (N = 28)	*p*-value
Age, years (mean, SD)	29.2 (9.32)	34.5 (12.6)	.25^b^
Gender			.46^c^
Male	4 (44.4)	11 (39.3)	
Female	4 (44.4)	16 (57.1)	
Transgender	0 (0)	0 (0)	
Other	1 (11.1)	1 (3.6)	
Not Reported	0 (0)	0 (0)	
Race			.30^c^
White/Caucasian	6 (66.6)	17 (60.7)	
Black/African American	1 (11.1)	6 (21.4)	
Hispanic/Latino	1 (11.1)	0 (0)	
Asian/Pacific Islander	1 (11.1)	0 (0)	
American Indian/Alaskan Native	0 (0)	1 (3.6)	
Multiple Races/Other	0 (0)	3 (10.7)	
Not Reported	0 (0)	1 (3.6)	
Education			.31^c^
Some High School	0 (0)	0 (0)	
High School Degree	3 (33.3)	6 (21.4)	
Some College	4 (44.4)	6 (21.4)	
College Degree	2 (22.2)	14 (50.0)	
Masters Degree	0 (0)	2 (7.1)	
Professional/Doctoral Degree	0 (0)	0 (0)	
Not Reported	0 (0)	0 (0)	
Psychiatric Conditions^d^			
ADHD	0 (0)	8 (28.6)	.16^c^
Alcohol Use Disorder	0 (0)	1 (3.6)	1^c^
Autism Spectrum Disorder	0 (0)	0 (0)	–
Bipolar Disorder	1 (11.1)	2 (7.1)	1^c^
Depression	5 (55.6)	15 (53.6)	1^c^
Generalized Anxiety Disorder	2 (22.2)	11 (39.3)	.45^c^
Obsessive Compulsive Disorder	0 (0)	4 (14.3)	.55^c^
PTSD	2 (22.2)	6 (21.4)	1^c^
Substance Use Disorder	0 (0)	1 (3.6)	1^c^

^a^Data are presented as N (%) unless otherwise specified.

^b^Statistical test: Independent samples t-test.

^c^Statistical test: Fisher exact.

^d^Based on self-report data.

**Figure 1:**
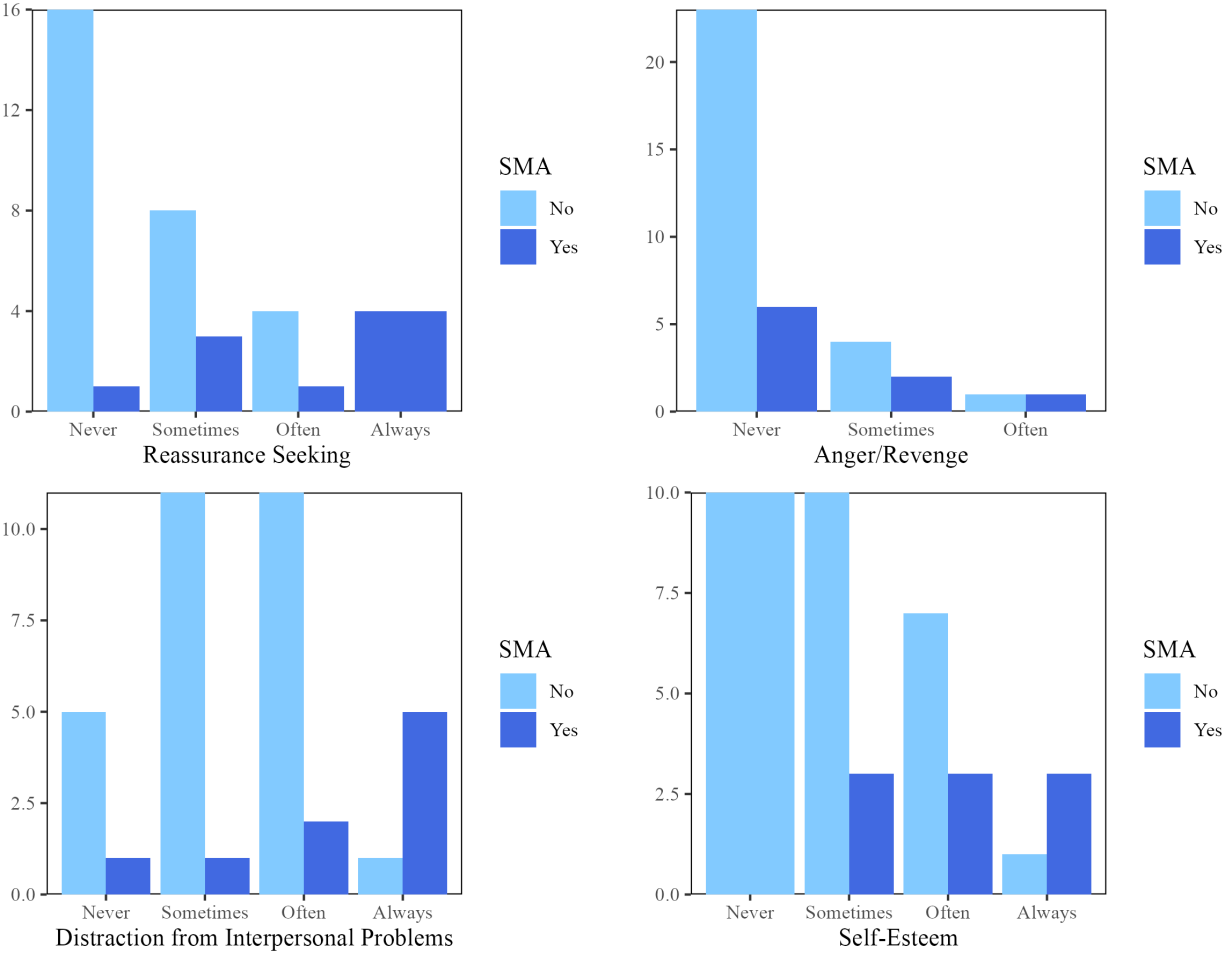
Frequency of Social Media-Related Behaviors in BPD+ SMA vs. BPD Only.

However, Mann Whitney U tests revealed that individuals with BPD meeting the criteria for SMA were more likely to report using social media for distraction from interpersonal problems (*U* =57, *p* =.01), self-confidence issues (*U* =54, *p* =.01), and reassurance seeking (*U* = 45.5, *p* =.003) as compared to individuals with BPD that did not meet criteria for SMA. There was no difference in the tendency to use social media for anger/revenge between individuals with BPD + SMA vs. those with only BPD (U = 102.5, *p* = .33) (**Figure 1**).

## Discussion

To our knowledge, this is the first study to investigate the prevalence of SMA among individuals meeting criteria for BPD. Consistent with our hypothesis, we found that individuals screening positive for BPD were more than 2 times more likely to meet criteria for SMA as compared to individuals who did not screen positive for BPD (25% vs. 9.6%). Interestingly, this rate of SMA in BPD subjects was also on the higher end of SMA rates reported in the literature to date (19–21), further suggesting that SMA may be particularly prevalent among individuals with BPD. We did find that subjects with BPD were younger and more likely to have comorbid depression than subjects in the control group. Therefore, one could argue that these variables contributed to the differences in SMA rates seen in this study, especially considering that SMA is associated with younger age and comorbid depression (35, 36). However, when we re-ran the analysis controlling for age and depression, the association between BPD and SMA still remained significant (see **Supplementary Appendix B**), suggesting that this finding is not due to between-group differences in either of these variables. For a condition that has traditionally been associated with comorbid substance use disorders, the finding that rates of SMA were elevated relative to controls makes an important contribution to burgeoning literature which suggests that behavioral addictions may also afflict individuals with BPD.

While this finding is important, what is perhaps more interesting is that consistent with our hypothesis, individuals with BPD meeting the criteria for SMA were more likely to report using social media for distraction from interpersonal problems and reassuring themselves that people still care about them, along with turning to social media when they have problems with their self-esteem or confidence, relative to individuals with BPD but no SMA. On the one hand, it is possible that engaging in these types of behaviors increases the risk that someone with BPD will develop SMA. The components model of addiction (37) suggests that mood modification is a key feature of addiction. Interpersonal difficulties and self-identity issues are core features of BPD (2), and individuals who turn to social media when these problems arise are ostensibly doing so to alter their mood or affect, through distraction, escape, reassurance, or other means. As such, individuals who use social media in this way may quickly develop an association between social media use and emotional changes, increasing the likelihood that an addiction will develop.

Similarly, with individuals posting many aspects of their lives on social media – from their whereabouts and activities to their emotions and life updates – social media provides an unparalleled opportunity to follow the activity of others. Fear of abandonment and the constant need to reassure oneself that others are still present and care about you are other hallmark features of the disorder (2). As such, it is perhaps unsurprising that we found that individuals with BPD reported using social media for reassurance purposes more than those without this condition do. If individuals with BPD learn that they can appease their fears of abandonment through social media use, they may start to engage in this behavior more regularly and become dependent on it, resulting in characteristics of SMA.

On the other hand, it is also possible that pre-existing SMA increases the likelihood that individuals with BPD will engage in the behaviors noted above. In other words, people with BPD may develop SMA because they possess other known risk factors, such as impulsivity, anxiety, rejection sensitivity or low self-esteem, to name a few (38–40); however, being dependent on social media provides ample opportunities for BPD symptoms to play out through these platforms. For example, for an individual who fears abandonment and spends a lot of their time on social media, it makes sense that social media may become a medium through which this symptom plays out. In this case, the tendency to use social media for BPD-like behaviors is not a cause of SMA, but a result of it. The directionality of this relationship cannot be determined from this cross-sectional survey (does SMA lead to BPD-like behaviors on social media or does using social media for BPD-like behaviors lead to SMA)? and is grounds for future research, but these finding do suggest that individuals with SMA are more likely to use social media for BPD-like behaviors.

Our findings may also be important for clinicians treating BPD. Approximately 1 in 4 individuals screening positive for BPD met criteria for SMA, suggesting that SMA is prevalent among individuals with this condition. Additionally, it is possible that social media use could worsen symptoms of BPD in some individuals. For example, although individuals may turn to social media for reassurance purposes, it does not mean that they will receive it. Research has shown that getting getter fewer-than-expected likes on a social media post is associated with a decreased sense of belonging and self-esteem (41). Similarly, ostracism on social media (e.g., being left “unread” on a social networking app, seeing posts about activities you were not invited to) negatively affects mood and even contributes to existential issues such as questioning your existence (42). These sorts of emotions are already highly prevalent among individuals with BPD (43), and social media may serve to worsen some of these issues.

Furthermore, social media is often a “highlight reel”, whereby many individuals only include information that is flattering or conveys some sort of success or prestige (44). This makes it all too easy for social media users to engage in upwards social comparison, which can result in reduced self-esteem (45). BPD is also associated with unstable self-esteem (46), and it is possible that social media use exacerbates this issue. Similarly, those with BPD often suffer from identity disturbances, part of which can be characterized by frequent fluctuations in goals and values (47). Regular use of social media exposes individuals to all sorts of activities, hobbies, or careers, along with a multitude of opinions and values. For someone who lacks a clear sense of who they are or what they believe, social media may contribute to frequent shifts in these constructs.

### Limitations

Despite the insights that this study provides, it is certainly not without its limitations. First, as the data was collected via an online survey, a self-report screen (MSI-BPD) was used to determine which subjects potentially met criteria for BPD. Although substantial research has shown that the MSI-BPD is valid and reliable scale (48) with good sensitivity and specificity for the diagnosis of BPD (32), it is not a gold-standard clinician diagnosis. As such, we cannot know whether the individuals who screened positive for BPD in this study would meet diagnostic criteria when assessed by a clinician. Alternatively, some scholars have proposed that a cutoff score of 7 is too high for the MSI-BPD and may therefore fail to detect individuals that meet criteria for this condition (48). As such, it may also be possible that using this cutoff score led to us identifying fewer individuals with BPD than existed in the sample. Therefore, future research should attempt to replicate these results with individuals who have a clinician-verified diagnosis of BPD.

Similarly, because the MSI-BPD was only a screen for BPD, we could not assess disorder severity in our sample. Therefore, we are unable to determine whether SMA is associated with greater severity of BPD symptoms. Our findings that individuals with BPD + SMA more frequently use social media for distraction from interpersonal problems, reassurance seeking, and self-confidence issues *may* indicate that these problems are more prevalent among those with SMA (and therefore represent individuals with more severe cases of BPD), but it may also just indicate that those with BPD + SMA engage in a greater *proportion* of their BPD-like behaviors on social media. Therefore, it is important that future research examines whether SMA impacts BPD severity using an established severity measure.

Next, while several studies have demonstrated that Prolific yields high data quality (49), participants on Prolific are not always representative of the population as whole or may be qualitatively different from other individuals. In our sample, we had a roughly representative mix of genders, but a majority of our subjects identified as Caucasian. Some research has also suggested that individuals who participate in research via online crowdsourcing platforms may have more mental health problems than the general population (50), which may have affected the rates of positive BPD screens seen in this study.

Finally, data regarding psychiatric conditions was done as a self-report. While we asked subjects what conditions they had been diagnosed with, there is no way to verify these diagnoses. As such, the rates of psychiatric conditions reported in this study may be over- or under-estimations.

### Future directions

Asides from addressing the limitations noted above, there are several important areas for future research. First, we did not investigate whether there are differences in rates of SMA based on the type of social media platform(s) used. Individuals do not engage with all social media platforms in the same way; some platforms, such as Instagram, Facebook, or TikTok, encourage users to post personal photos and videos while other platforms, such as Pinterest, encourage users to browse and share pre-existing materials. If the opportunity to view and interact with the personal content of others contributes to SMA in those with BPD, perhaps those who use social media platforms that better cater to these behaviors would be more likely to develop SMA.

Next, BPD is often described as a heterogenous disorder (51) with different subtypes (see 52 for a review); for example, some research has suggested that individuals fall into an *instability* subtype or an *interpersonal disturbance* subtype (53). We found that SMA was positively associated with using social media for distraction from interpersonal problems, reassurance seeking, and self-confidence issues, all of which seemingly fall under behaviors of an *interpersonal disturbance* subtype. Additionally, we found that SMA was *not* associated with anger/aggression on social media, a behavior that may fall into the *instability* subtype. As such, perhaps those falling into the *interpersonal disturbances* subtype would be more like to meet the criteria for SMA than individuals falling into the *instability* subtype. However, more research is certainly needed to substantiate this hypothesis.

## Conclusions

To our knowledge, this is the first study to demonstrate that SMA is more common among individuals screening positive for BPD relative to the general population. Although SMA has yet to be recognized as an official disorder, these results suggest that clinicians should still be aware of SMA among their BPD patients, especially as individuals with SMA were more likely to engage in potentially maladaptive behaviors, such as using social media for distraction from interpersonal problems, reassurance seeking, and self-confidence issues. If social media is a medium through which BPD symptoms are expressed, it is important for clinicians to be aware of this. However, whether addressing SMA among individuals with BPD may provide relief from BPD symptoms itself has yet to be seen, but future research would be wise to investigate this possibility.

## Data Availability

The raw data supporting the conclusions of this article will be made available by the authors, without undue reservation.
