# Supplementary material for: Social media addiction and borderline personality disorder: a survey study
Source: Front Psychiatry. 2025 Jan 8;15:1459827. doi: 10.3389/fpsyt.2024.1459827 (PMC11751677; doi:10.3389/fpsyt.2024.1459827)
Supplement: Supplementary file 1 [file Table1.docx]

**Supplemental Appendix A**

Table 1. Social Media Platforms in BPD and Controls^a^

|  | **Total (N = 280)** | **BPD (N = 37)** | **Controls (N = 243)** | ***p*-value^b^** |
| --- | --- | --- | --- | --- |
| Instagram | 183 (65.3) | 25 (67.8) | 158 (65.0) | .73 |
| Facebook | 188 (67.1) | 21 (56.8) | 167 (68.7) | .17 |
| X (Formerly Twitter) | 141 (50.4) | 21 (56.8) | 120 (49.4) | .39 |
| Tiktok | 141 (50.4) | 27 (73.0) | 114 (46.9) | .003 |
| Snapchat | 79 (28.2) | 17 (45.9) | 62 (25.5) | .01 |
| Pinterest | 82 (29.2) | 16 (43.2) | 66 (27.1) | .04 |
| Reddit | 188 (67.1) | 25 (67.6) | 163 (67.1) | .92 |
| ^a^All data are presented in N(%) format  ^b^Chi-square tests were used for all comparisons | | | | |

**Supplemental Appendix B**

Table 1. Logistic Regression Predicting SMA (Yes vs. No)

| Predictor | B | SE (B) | Odds Ratio | 95% CI | *t* | *p*-value |
| --- | --- | --- | --- | --- | --- | --- |
| Intercept | .26 | .06 | 1.30 | 1.15, 1.47 | 4.25 | <.001 |
| Age | -.004 | .002 | .995 | .992, .998 | -3.01 | .003 |
| MDD | .04 | .41 | 1.04 | .96, 1.23 | .96 | .34 |
| BPD | .12 | .058 | 1.13 | 1.01, 1.26 | 2.10 | .037 |

The results of the logistic regression reveal that a positive screen for BPD (based on the MSI-BPD) increases the odds of meeting criteria for SMA, even after controlling for age and a MDD diagnosis.
